# Reliability of Publicly Available Digital Endoscopes in Otolaryngology: A Pilot Study

**DOI:** 10.1097/ONO.0000000000000055

**Published:** 2024-05-17

**Authors:** Michael P. McWilliams, Kevin Quinn, Lawrance Lee, Nauman F. Manzoor, Daniel H. Coelho

**Affiliations:** 1 Department of Otolaryngology – Head & Neck Surgery, Virginia Commonwealth University School of Medicine, Richmond, VA.

**Keywords:** Consumer electronics, digital otoscopy, telemedicine

## Abstract

**Objective:**

To compare the utility, accuracy, and confidence of an inexpensive, commercially available endoscope (not specifically designed for medical use) with traditional methods in the diagnosis of otologic conditions.

**Study Design:**

This is a prospective study.

**Methods:**

Following institutional review board approval, patients were recruited from a tertiary university-based otology/neurotology clinic. Complete history and physical were obtained by the resident, including both traditional handheld otoscopy and image captured from a commercially available digital endoscopic device (AnyKit Digital Otoscope with 4.5-inch screen). The patient was then presented to the attending without the endoscopic images and a putative diagnosis was made. The endoscopic images were then shown and the putative diagnosis was affirmed or rejected. The attending then examined the patient and determined the final diagnosis using the microscope. Data collected included resident year, resident and attending diagnosis before and after digital-otoscopic images, confidence in diagnosis (1–5 scale), and agreement between the initial putative diagnosis and the final diagnosis. Noninferiority testing was calculated using inter-rater agreement between digital-otoscopic and final diagnoses. Differences between resident and attending confidence were analyzed. A power analysis was performed and the sample size was calculated a priori.

**Results:**

A total of 62 participants (114 ears examinations) were enrolled. Cohen’s kappa coefficient showed very high agreement between both resident and attending digital-otoscopic and final diagnosis (kappa = 0.868 and 0.882, respectively) suggesting noninferiority between the digital otoscope and the final diagnosis. There was no significant difference between attending confidence in diagnosis following resident presentation versus attending confidence in diagnosis after reviewing images (4.65 vs 4.61, *P* = 0.701). Average resident confidence in digital-otoscopic diagnosis remained above 4.2 throughout the study.

**Conclusion:**

Inexpensive and readily available digital endoscopes are not inferior to the traditional methods of resident-attending consultation and may provide some substantial benefits. Such devices have the potential to enhance both patient care and resident education when faculty are not immediately available (ie, inpatient and emergency room consults) and improve patient-initiated communications.

With technological improvements over the past several years, there has been substantial growth in the market for consumer electronics and health information products that patients can access/obtain within the comfort of their own home (1–3). Specific to the field of otology, this technological improvement has led to the emergence of high-quality commercially available digital endoscopes (CAE). Contrasted with medical grade digital otoscopes that are typically very expensive and not available to the public, CAE are relatively affordable and often marketed to consumers as a tool for cerumen removal. These devices have gained popularity in recent years due to their ability to display the ear in real time to users, as well as their zoom and image-capturing capabilities. Though there is currently limited use in academic and medical settings, the low price and wide availability of CAE (to the general public, patients, and clinicians alike) make them an interesting alternative to the standard bedside otoscopic evaluation.

Following the emergence of the COVID-19 pandemic, telemedicine rapidly became a ubiquitous component of the global healthcare system with the number of US physicians using telehealth more than doubling since 2019 (4). This trend has led to an increased need for digital communication with patients, which is particularly difficult in the field of otolaryngology given that much of the diagnostics require examination of areas that are difficult to access and visualize. CAE have the potential to help fill this need. Additionally, this technology has the potential to expedite communication between residents and attending physicians in an academic setting, aiding both patient diagnosis and trainee education.

While current research and anecdotal experience from physicians show that this technology in the hands of untrained patients/parents is not consistently useful (5,6), prior studies examining the ability of physicians to implement smartphone CAE have shown enough promise to suggest that the CAE could be a useful tool for patient-physician or primary care-specialist communication (6,7). Before the widespread adoption of CAE in inpatient and emergency settings, additional data on reliability, ease of use, and validation are needed. The aim of this study is to compare diagnostic accuracy of a CAE with that of traditional diagnostic techniques, while also evaluating the learning curve and user comfort/confidence with CAE in the outpatient clinic setting prior to more generalized use.

## METHODS

**FIG. 1.**
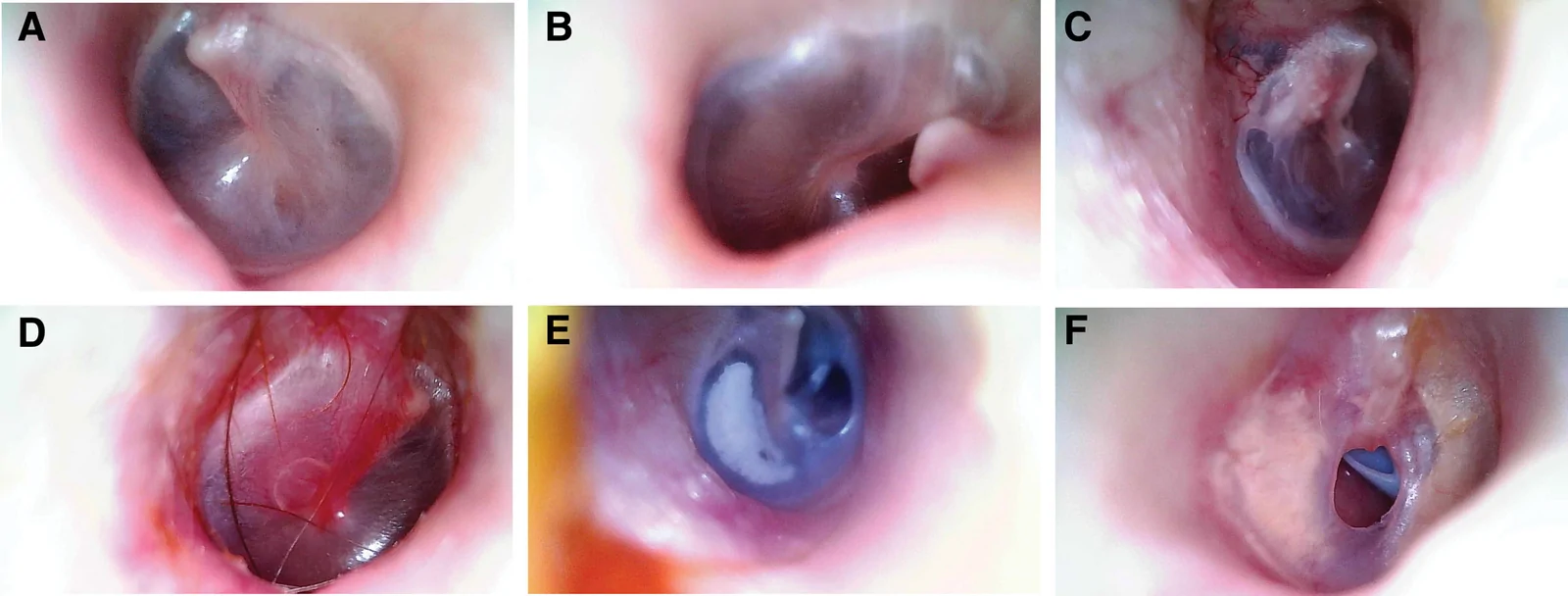
Examples of image quality and various diagnoses made. *A*, Normal TM exam. *B*, Proximal EAC exostosis. *C*, Intact TM with healed postsurgical changes. *D*, Glomus tympanicum tumor. *E*, Myringosclerosis. *F*, Perforated TM with PE tube in the middle ear space. TM indicates tympanic membrane.

Approval for the study with human subjects was obtained from the Virginia Commonwealth University Institutional Review Board—IRB protocol number: HM20014233 (principal investigator: D.H.C.). This prospective pilot study began by trialing multiple CAE, not to be confused with more expensive otoendoscopes designed exclusively for use in medical settings. These devices were readily available for purchase online (amazon.com)—the majority of which cost between USD 50–90. CAE evaluated primarily fell into 2 categories: those that required wired connection to smartphones and those having a built-in display screen. The AnyKit Digital Otoscope with a built-in 4.5-inch screen was ultimately selected for this study based on subjective evaluation of image quality, ease of use, and affordability (Fig. 1).

After informed consent was obtained, patients in the otology/neurotology clinic had a complete history and physical obtained by a resident physician. This history and physical included both traditional handheld otoscopy and image captured with the CAE. The patient was then verbally presented to the attending without CAE images and a putative diagnosis was made. The CAE images were then shown to the attending physician and the putative diagnosis was affirmed or rejected. The attending then personally examined the patient and determined the final diagnosis using the microscope. Throughout this process, data were collected on resident year, resident bedside otoscopic diagnosis, resident diagnosis with CAE images, attending diagnosis based on resident verbal presentation, attending diagnosis from CAE images, and final attending diagnosis upon microscopic evaluation. Confidence level, graded on a 1–5 scale (1, not at all confident to 5, very confident), was obtained from both resident and attending physicians at each diagnosis. CAE were sterilized using alcohol swabs between all patients.

A power analysis with sample size calculation was performed based on previous studies showing that the typical diagnosis accuracy rate for otolaryngologists with a standard bedside otoscope is approximately 75%–85% (8,9). Using 85%, with the goal of illustrating that commercially available endoscopes are noninferior, an anticipated diagnostic accuracy of 75% using CAE (within 10% of comparison) was considered clinically equivalent. Power was set to 0.8. Dichotomous endpoint, one-sample sample size calculation yielded a necessary sample size of 113 ears. Agreement between methods of diagnosis and final diagnosis was calculated via Cohen’s kappa test (10). Resident confidence over time was analyzed using mean and standard deviation. Differences in diagnosis confidence were calculated using analysis of variance testing and *t* tests. All analysis was done in either R 4.1.1 or SPSS Statistics (version 28.0.1.0; IBM Corp, Armonk, NY).

## RESULTS

A total of 62 patients and 114 ear examinations were included in the study. Diagnoses were made by 7 otolaryngology residents ranging from PGY 1–5 and 2 attending neurotologists. About 35 unique diagnoses were made.

Table 1 lists the Cohen’s kappa coefficients for each method of diagnosis, a measure of agreement with the final diagnosis made via attending microscopic examination. Attending diagnoses following resident verbal presentation had the highest kappa value of 0.919. Resident diagnoses made with a traditional otoscope had a kappa value of 0.879. Resident and attending diagnoses made upon evaluation of CAE images had kappa values of 0.868 and 0.882, respectively (no statistically significant difference, *P* = 0.397).

**TABLE 1.** Agreement with final diagnosis expressed via Cohen’s kappa coefficient (N = 114)

Method of diagnosis	Cohen’s kappa coefficient	Asymptotic standard error
Resident otoscope diagnosis	0.879	0.037
Resident CAE diagnosis	0.868	0.039
Attending diagnosis from resident description	0.919	0.031
Attending CAE diagnosis	0.882	0.037

CAE indicates commercially available digital endoscopes.

There was no statistically significant difference in diagnostic agreement between a standard bedside otoscopic examination and CAE examination (*P* = 0.418). Resident physician confidence in diagnoses made with the CAE was equal to confidence in bedside otoscopic examination. Additionally, there was no statistically significant difference in attending confidence in resident presentations with and without CAE images (*P* = 0.964). These data suggest that patient examination with CAE is noninferior to traditional bedside otoscopic examination.

Diagnostic confidence levels were tracked longitudinally throughout the study (grouped by month). Mean resident confidence with the CAE remained above 4.5/5 for the duration of the study. Table 2 shows the mean and standard deviation of the confidence levels associated with each method of diagnosis. Analysis of variance testing looking at possible differences between groups yielded a *P* value of 0.538—suggesting no significant difference between confidence in the method of diagnosis.

**TABLE 2.** Confidence level by method of diagnosis and analysis of variance significance value (N = 114)

Method of diagnosis	Mean confidence (1–5 scale)	Standard deviation
Resident otoscope diagnosis	4.518	0.801
Resident CAE diagnosis	4.518	0.924
Attending diagnosis from resident description	4.649	0.624
Attending CAE diagnosis	4.601	0.918
*P* value	0.538	

CAE indicates commercially available digital endoscopes.

There were no patient injuries/burns associated with the use of the device throughout the study.

## DISCUSSION

The COVID-19 pandemic and the associated rise of telemedicine have provided the impetus for the development of technology that facilitates at-home patient examination and assessment. The current study attempts to better understand the capability of current technology regarding the potential for remote otoscopic examination and evaluate how this technology can further both patient care and medical education. These results demonstrate that diagnoses made by both resident and attending physicians using images from our CAE have strong agreement with the final diagnosis made via microscopic examination, supporting the hypothesis that the CAE are noninferior to traditional bedside otoscopic examination. This finding is in keeping with current literature, as several papers have shown that, in the hands of trained providers, video-otoscopy can serve as a reliable means of diagnosis. Studies by Shah et al (6) and Mandavia et al (7) evaluating smartphone otoscopy, rather than CAE with a built-in display, found that physicians were ~90% accurate in diagnoses made with a smartphone otoscope and that these diagnoses agreed with pneumatic otoscopy findings (kappa value of 0.71). Additionally, a 2021 systematic review on video-otoscopy by Metcalfe et al (11) showed that pooled sensitivity and specificity for CAE otoscopy were 0.81 and 0.95, respectively. Notably, however, the present study demonstrates noninferiority using a relatively cheap CAE ($80) marketed to the general public, while devices included in the above articles and systematic review are endoscopes designed specifically for medical use that come at a higher cost (ranging from $250 to greater than $1500).

Both resident and attending physician confidence in diagnoses made with the CAE remained high (>4.2 of 5) throughout the duration of the study, suggesting an easy learning curve for mastery of the device. Anecdotal experiences from residents and students involved in the study noted that the CAE are often easier to maneuver and provide better visualization of the tympanic membrane than traditional bedside otoscopes. This is supported by a 2016 study by Sahyouni et al (12) showing that an iPhone otoscope was preferred to a traditional otoscope by all stages of medical providers.

The above data demonstrate that CAE are both easy to utilize and agree well with both traditional otoscopic examination and microscopic examination. They also exhibit that the potential CAE have to expand the availability of specialist evaluation of otologic examinations and ultimately improve both patient care and medical student/resident education. An example of this impact is found within the field of dermatology, which has shown the benefit of using images in documentation to aid in patient care and peer communication (13–15). One study by Kvedar et al (16) even found that digital images could serve as a substitute for physical examination in greater than 80% of their cases. It is reasonable to expect that these findings would transfer well to the field of otology. With regard to patient care in an academic setting, there is added utility for CAE in situations where faculty may not be readily available such as nights, weekends, off-site patient visits, or emergency department consults. Resident physicians or medical students could capture high-quality digital images and rapidly share them with faculty when discussing patients. This is a potential improvement over verbalizing physical exam findings on the phone, as it allows for bypassing some knowledge limitations of junior providers.

In addition, there is potential to advance otologic care for patients in remote locations and with limited access to health care. Rural providers will be able to utilize these devices and consult otolaryngologists with images—increasing access to subspecialty care. Similar findings are widely found in current literature. Teleconsults have proven to be remarkably effective in several other fields and have been found to save time, reduce expenses, and improve communication between patients and providers in rural communities (16,17). Totten et al’s (18) 2022 systematic review of 166 studies found that telehealth patient outcomes in multiple settings (outpatient, inpatient, and emergency care) were similar to that of in-person care but were far more accessible to rural residents. Many studies cite high start-up costs and provider reluctance as barriers to widespread implementation of this model, but recent improvements in telemedicine infrastructure and decreasing cost of technology, especially with regard to otoscopic evaluation, have lowered this hurdle. Finally, the ability to review images and suggest treatment remotely will likely reduce the burden of work providers currently face from consults.

Furthermore, there is a capacity to improve medical student/resident education via the integration of CAE into the medical curriculum. Prior studies looking at similar products have shown that the use of digital otoscopes led to increased accuracy of ear examinations performed by trainees (12,19). A device with an easy learning curve that can be used on oneself, peers, or patients allows trainees to better familiarize themselves with ear anatomy and pathology—which may aid in the mastery of traditional otoscopic examination.

An unforeseen benefit that was found during the study is that many patients preferred reviewing the image findings with their providers in real time. Several patients reported a high degree of interest in their images and enjoyed being involved with their personal care—a result that past studies show improves patient outcomes and satisfaction (20). Anecdotal experiences of the authors further suggest improved patient understanding of their pathology associated with image review.

**FIG. 2.**
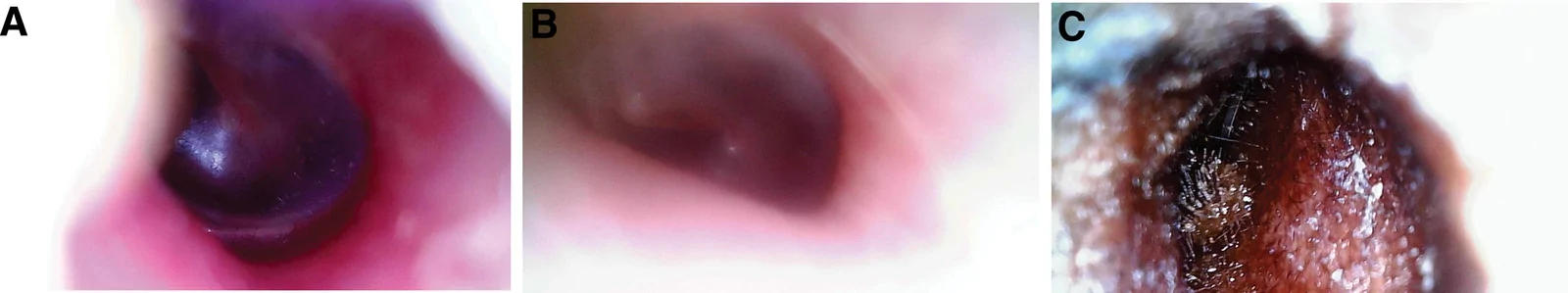
Demonstration of implementation challenges. *A*, Canal wall shadowing in a normal ear. *B*, Clouding of lens by cerumen. *C*, Cerumen impaction obstructing view.

Two major implementation challenges arose associated with device use. First, we encountered difficulty with shadowing from the medial external auditory canal in patients with more tortuous canals. This shadow, if unrecognized, could be mistaken for pathologies such as hemotympanum. Additionally, several residents noted that the light used in the device is cooler (more blue/white) than the light typically used in a standard otoscope. The greatest impedance to obtaining high-quality images was the presence of cerumen in the canal obstructing the view or clouding the lens and field of view. Several patients required cerumen removal prior to proper examination. Both difficulties have been echoed by similar studies in the past, and future work should be done to try and resolve them (11). Examples of these difficulties can be seen in Figure 2.

This study is not without its limitations. First, it was performed at a single institution with 2 attending neurotologists and 7 otolaryngology resident physicians. The device being utilized and evaluated by a limited number of reviewers could impact generalizability of the findings. Further, the pictures used were taken by individuals with anatomic training and it is unclear if patients attempting to communicate with their doctor would be able to obtain images of sufficient quality. Additionally, the study reviewed only one of many digital otoendoscopes currently available on the market. There is likely substantial variation in reliability and image quality between products. Finally, while we experienced no issues with regard to safety/patient outcomes with the CAE, it is not at this time an FDA-approved device. Nonetheless, the current study provides clinically significant information about the utility of this CAE and its potential to improve patient care, trainee education, and patient-related/initiated communications.

Further studies are needed to assess whether digital otoendoscopes are superior to standard bedside otoscopy. While there were no issues with patient injury or infection in our study, additional research could be done to determine best practices regarding cleaning/hygiene between patients. An alternative research avenue could assess the utilization of artificial intelligence to analyze photographs taken with CAE and make diagnoses. Recent studies have shown the success of artificial intelligence driven image interpretation in the fields of dermatology and radiology, and it is likely that this technology could be adapted to the field of otolaryngology as well (21,22).

## CONCLUSION

This study assesses the capability of a commercially available endoscope to assist in patient care and trainee education. The data show that diagnoses made via both bedside otoscopic exam and CAE use had high agreement with final diagnoses made via attending microscopic examination. While the CAE used is not yet FDA approved, these results suggest that CAE exam is noninferior to bedside otoscopic exam while offering an easy learning curve to trainees. This highlights the potential utility CAE have for improving patient care, trainee education, and patient-initiated communication. Studies evaluating the practical utility of these devices for inpatient and emergency room consults are ongoing.

## FUNDING SOURCES

This study was generously supported through a grant from the MEDRVA foundation.

## CONFLICT OF INTEREST STATEMENT

None declared.

## DATA AVAILABILITY STATEMENT

Datasets generated and analyzed during the current study are available upon request of the author.
